# Supplementary material for: Screening and Identification of Novel Soluble Epoxide Hydrolase Inhibitors from Corn Gluten Peptides
Source: Foods. 2022 Nov 18;11(22):3695. doi: 10.3390/foods11223695 (PMC9689838; doi:10.3390/foods11223695)
Supplement: Supplementary file 1 [file foods-11-03695-s001.zip › foods-1990559-supplementary.pdf]

Supplementary Materials

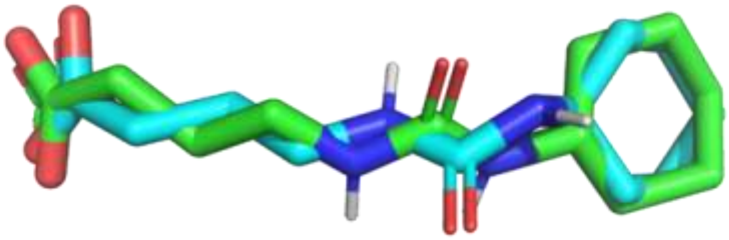

Figure S1. The alignment between MOE docked pose (cyan) and native pose (green).

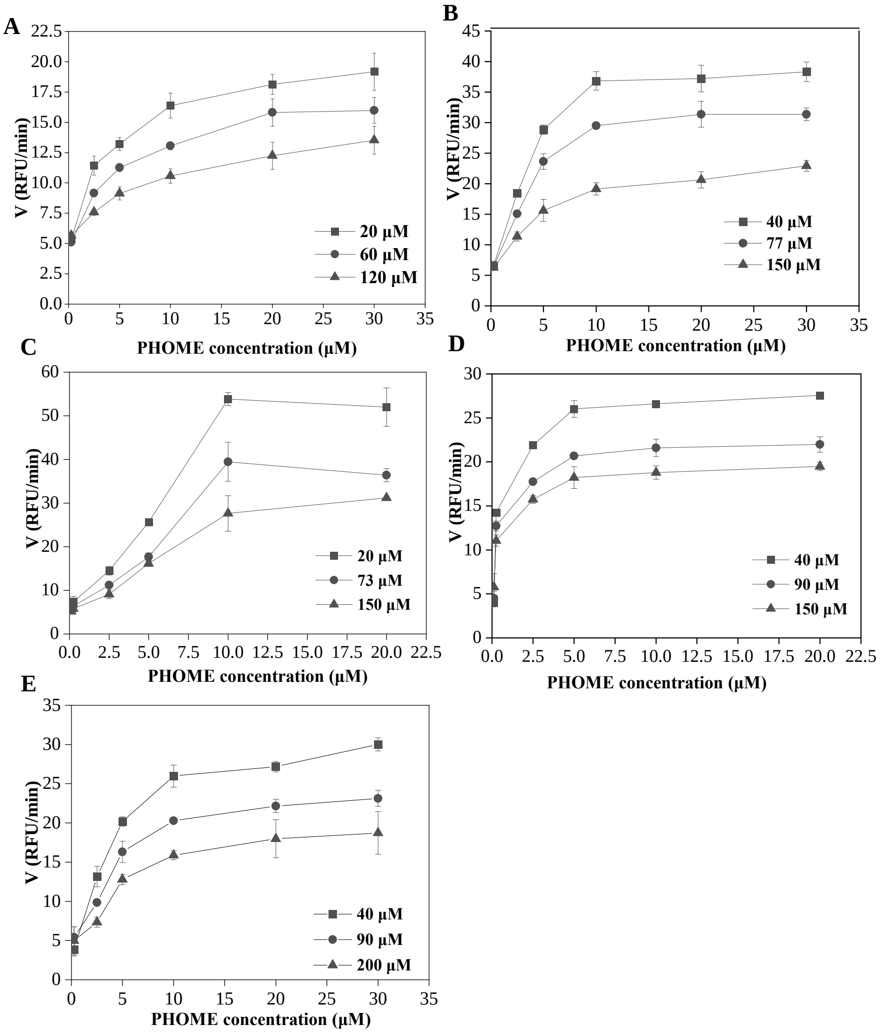

Figure S2. Inhibition kinetics of peptides WEY (A), WWY (B), WYW (C), YFW (D),and YFY (E) on sEH.

Table S1: Physicochemical properties and toxicity of sEH inhibitory peptides.

No.	Sequence	MW (Da)	pI	Net charge at pH 7	Water solubility	Toxicity
1	WEY	496.52	0.95	-1	good	non-toxic
2	WYW	553.61	3.44	0	poor	non-toxic
3	YFW	514.57	3.31	0	poor	non-toxic
4	WWY	553.61	3.51	0	poor	non-toxic
5	YFY	491.53	3.37	0	poor	non-toxic
